# Supplementary figures and images for: Finding and extending ancient simple sequence repeat-derived regions in the human genome
Source: Mob DNA. 2020 Feb 17;11:11. doi: 10.1186/s13100-020-00206-y (PMC7027126; doi:10.1186/s13100-020-00206-y)

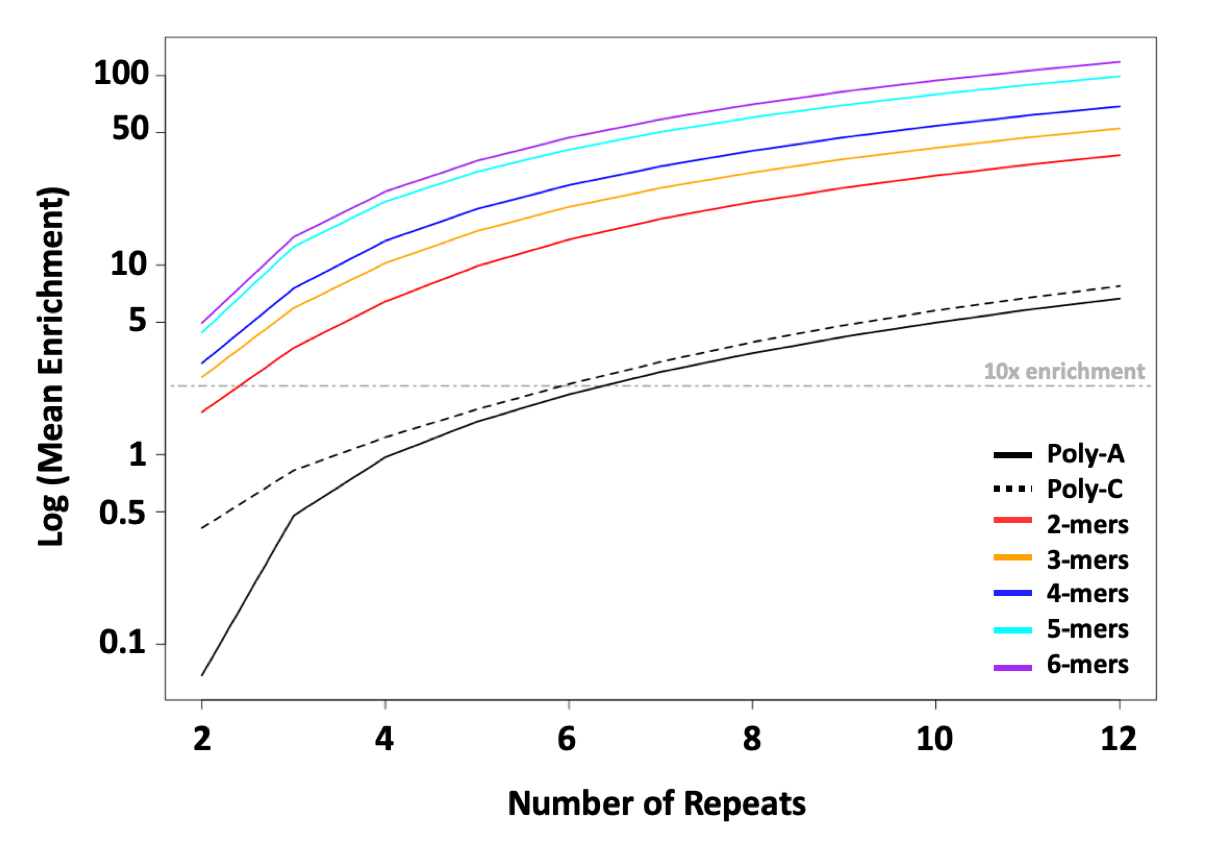

Supplement: Supplementary file 1 — Additional file 1: Figure S1. Enrichment of SSRs in the human genome. The mean enrichment of perfect repeats is shown relative to expectation from single nucleotide frequencies. All SSR motifs of a given length were clustered into groups, except that the Poly-A and poly-C single nucleotide repeats are shown as separate lines. The enrichment is shown for the number of repeats of a given size observed in tandem, and the gray dashed lines indicate 10x, 100x, and 1000x enrichments. [file 13100_2020_206_MOESM1_ESM.png]

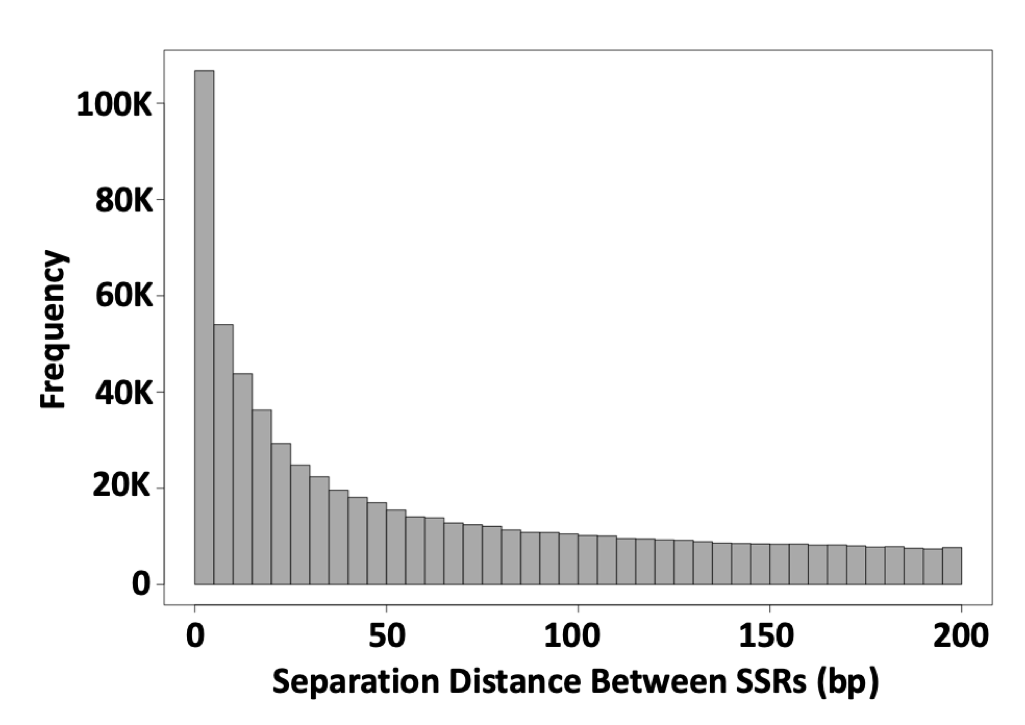

Supplement: Supplementary file 2 — Additional file 2: Figure S2. Separation distance between perfect SSRs in the human genome. The frequency of pairs of perfect SSRs ≥12 bp long with a given separation distance is shown. The separation distances were binned into groups of 5. The results in A) are for a masked version of the human genome, while B) shows results for an unmasked genome, demonstrating the strong effect and particular features of transposable element SSRs. [file 13100_2020_206_MOESM2_ESM.png]

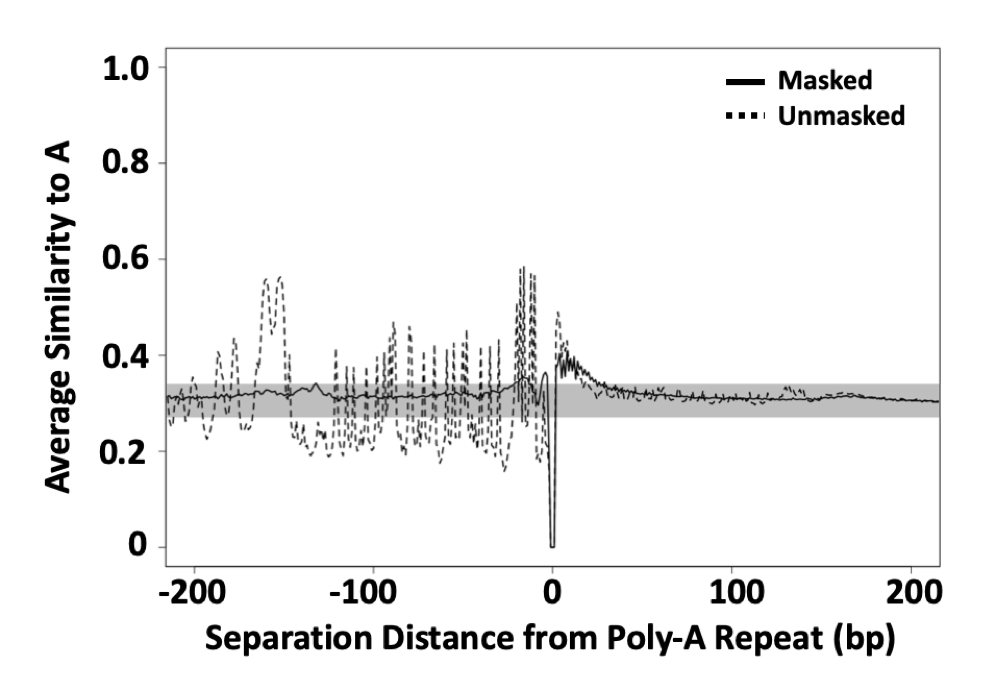

Supplement: Supplementary file 3 — Additional file 3: Figure S3. Asymmetric similarity to poly-A. The frequency of adenine nucleotides (A) at every site within 200 bp of perfect poly-A repeats. The solid line shows the frequency of A in a human genome where all transposable elements have been masked and the dotted line shows the frequency in an unmasked human genome. As a reference, the gray box represents a range of 3 standard deviations from the mean frequencies of A calculated in 700 bp windows from 300 to 1000 bp away from both ends of all perfect repeats. The strongly varying frequencies in the unmasked genome are mostly a symptom of the high copy number of retroelements such as Alu and Line1. The asymmetric frequency of A’s adjacent to perfect A repeats in the masked genome likely reflects incomplete masking of transposable elements and the existence of other unmasked retrotransposed sequences in what would have been the 5′ region of the retrotransposed poly-A mRNAs. [file 13100_2020_206_MOESM3_ESM.png]

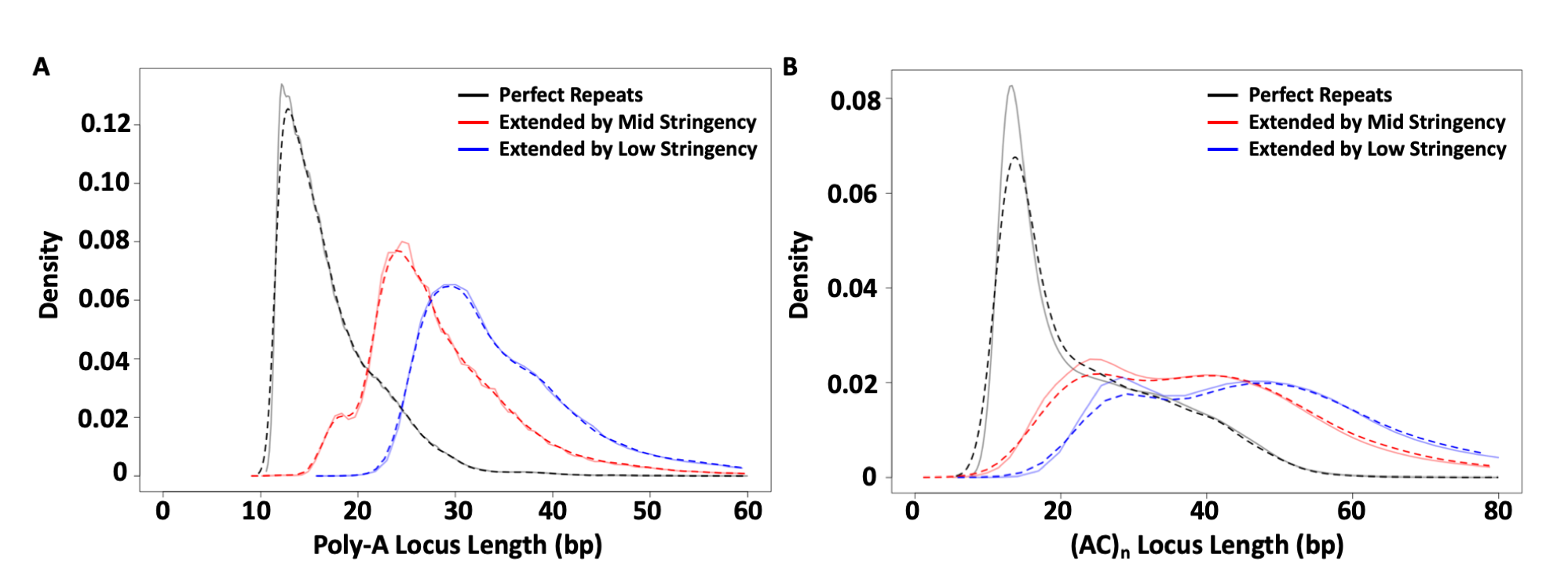

Supplement: Supplementary file 4 — Additional file 4: Figure S4. Cloud extension length distributions of training and test loci. Locus length density plots of SSR loci containing perfect repeats (black) and lengths after extension by mid- (red) and low-stringency (blue) cloud sets. Solid lines depict the distributions of lengths for training loci and dashed lines depict the almost perfectly overlapping distributions of lengths for test loci. [file 13100_2020_206_MOESM4_ESM.png]

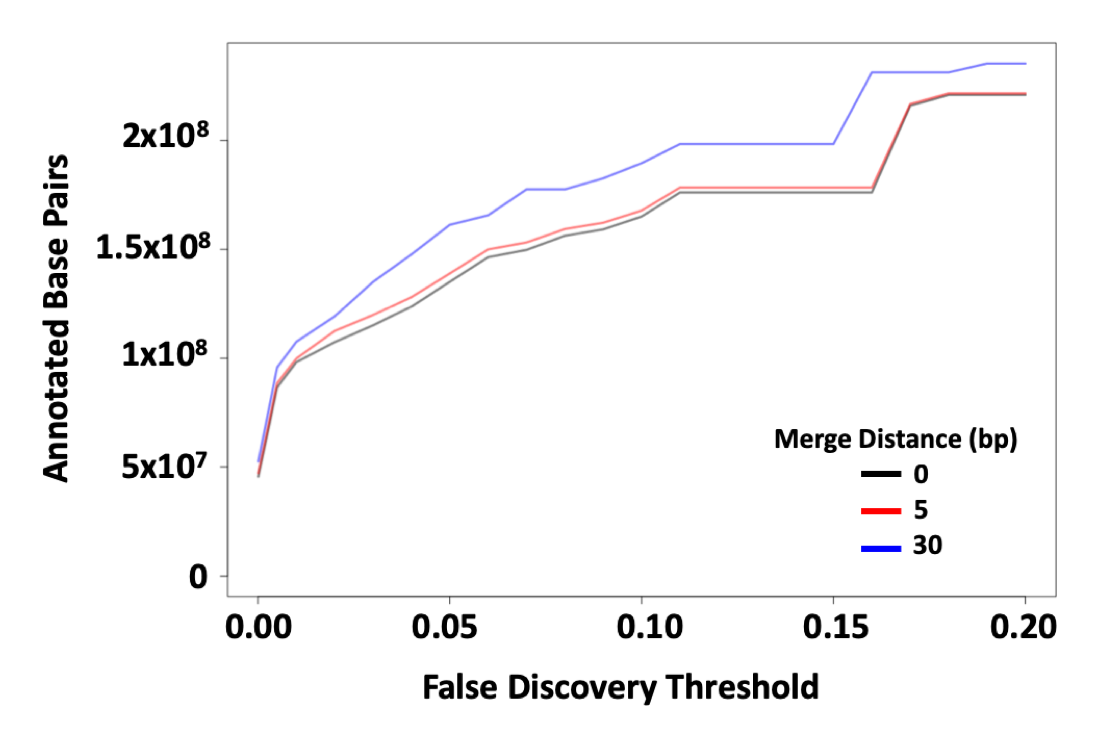

Supplement: Supplementary file 5 — Additional file 5: Figure S5. Genomic SSR content annotated with different merge distances and false discovery thresholds. The number of bp in the human genome that were annotated by SSR-clouds under various conditions are shown. With different merge distances and false discovery thresholds. Three lines are shown for merge distances of 0 bp (black), 5 bp (red), and 30 bp (blue), with the per-locus maximum false discovery criterion on the X axis. [file 13100_2020_206_MOESM5_ESM.png]
